# Coordination Nanosheets Stabilizing Efficient Tin-Based Perovskite Solar Cells

**DOI:** 10.1021/acsami.5c05011

**Published:** 2025-04-24

**Authors:** Dhruba B. Khadka, Yan-Chen Kuo, Yi Zhen Li, Muhammad Waqas, You-Jia Xu, Masatoshi Yanagida, Hiroshi Nishihara, Kazuhito Tsukagoshi, Mitch M. C. Chou, Yasuhiro Shirai, Ying-Chiao Wang

**Affiliations:** † Photovoltaic Materials Group, Center for GREEN Research on Energy and Environmental Materials, National Institute for Materials Science (NIMS), 1-1 Namiki, Tsukuba, Ibaraki 305-0044, Japan; ‡ Department of Materials and Optoelectronic Science, National Sun Yat-sen University, Kaohsiung 804, Taiwan, R.O.C.; § Research Institute for Science and Technology, Tokyo University of Science, 2641 Yamazaki, Noda, Chiba 278-8510, Japan; ∥ WPI International Center for Materials Nanoarchitectonics (WPI-MANA), National Institute for Materials Science (NIMS), 1-1 Namiki, Tsukuba, Ibaraki 305-0044, Japan; ⊥ Academy of Innovative Semiconductor and Sustainable Manufacturing, National Cheng Kung University, Tainan 70101, Taiwan, R.O.C.

**Keywords:** tin perovskite, coordination nanosheet, crystallization, oxidation, additive

## Abstract

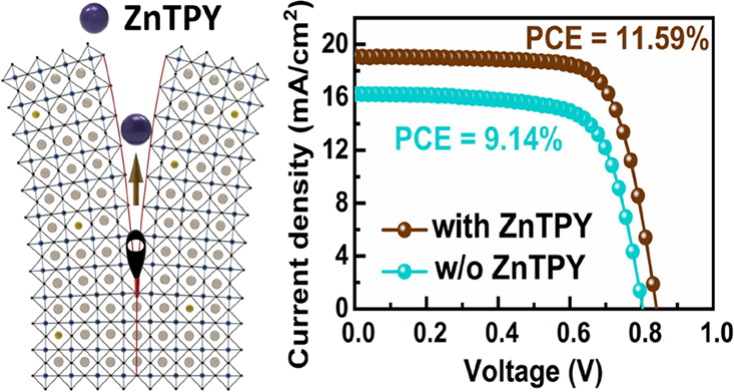

Tin-based perovskites, characterized by their advantageous bandgap and much lower toxicity, have emerged as a promising alternative to lead-based perovskites in solar cell applications. However, the efficiency and stability of tin-based perovskite solar cells (Sn-PSCs) are still limited by defects resulting from the easy oxidation of Sn^2+^ to Sn^4+^. Herein, an approach to enhance the optoelectronic performance of Sn-PSCs by incorporating terpyridine-zinc(II) (ZnTPY) coordination nanosheets (CONASHs), synthesized via liquid–liquid interfacial polymerization, into tin-based perovskites is delivered. Following physical fragmentation, ZnTPY CONASHs, enriched with unsaturated terpyridine groups, undergo multidentate chelation with SnI_2_, forming ZnTPY:SnI_2_ heterogeneous nuclei. This process effectively enhances the crystallization of tin-based perovskites while mitigating recombination and defect chemistry related to Sn^2+^ oxidation. As a result of superior crystal quality, the ZnTPY CONASHs-modified tin perovskite exhibits a longer photoluminescence lifetime. Consequently, the Sn-PSC incorporating ZnTPY complex achieves a power conversion efficiency of 11.59%, compared to 9.14% for the control device, along with improved operational stability with encapsulation. Thus, this work underscores the critical role of coordination nanosheets for regulating coordination in the precursor solution to achieve high-quality tin-based perovskite films, offering a pathway to more efficient and stable Sn-PSCs.

## Introduction

Lead-incorporated halide perovskite crystals have made significant advancements within the photovoltaic community, leading to an increase in the power conversion efficiency (PCE) of perovskite solar cells (PSCs) to over 26% in the past decade.^1−4^ However, the well-established environmental and human health risks linked to lead pose a substantial barrier to the commercialization of these lead-containing photovoltaic technologies.^5,6^ Therefore, several low-toxicity elements, such as copper,^7^ bismuth,^8^ and tin (Sn),^9^ have been explored as potential alternatives to highly toxic lead. Among these candidates, Sn-related PSCs (Sn-PSCs) are recognized as the most suitable substitute for lead PSCs, owing to their ideal bandgaps of 1.2-l.4 eV,^9^ approaching the Shockley-Queisser limit,^10^ as well as their high carrier mobility,^11^ high absorption coefficient,^12^ and low exciton binding energy.^13^ However, the PCEs of Sn-based PSCs remain considerably lower than those of lead-based PSCs due to several defect-induced factors.^14^ The primary defect in Sn-perovskite crystals arises from the easy oxidation of Sn^2+^ to Sn^4+^, which leads to the generation of numerous Sn^2+^ vacancies.^15,16^ These point defects induce p-type doping, creating multiple trap-state densities that ultimately reduce carrier diffusion lengths.^17^ Additionally, the rapid crystallization of SnI_2_ with organic ammonium ions complicates the control of film morphology during solution processing,^18^ resulting in severe nonradiative recombination losses^19^ and further limiting the performance of Sn-PSCs.

To address these issues, substantial efforts have been made in recent years. For instance, mixed-organic-cation engineering has been proven to be an effective strategy for modulating crystallization and film morphology in Sn-PSCs.^20,21^ Initially, methylammonium cation (MA^+^) was proposed as the sole organic cation for Sn-PSCs^22,23^; nevertheless, because of the deficient quality of tin perovskite films, the first-generation devices were unstable and exhibited poor reproducibility.^24^ Considering this challenge, partially replacing MA^+^ with formamidinium cation (FA^+^) at the A-site of perovskites would be more advantageous, as the FA^+^ provides better stabilization of the crystal structure compared to MA^+^ in tin-based perovskite crystals.^25^ This occurs because the larger size of FA^+^ reduces the antibonding coupling of Sn s orbitals with I p orbitals in Sn-PSCs.^26−28^ Thus, the formation energy of tin vacancies in Sn-based perovskite crystals increases. Consequently, the charge carrier type of FA^+^-included tin perovskite can be modified from p-type to an intrinsic state, indicating that point defects can be considerably mitigated. While reducing point defects can enhance PCEs of Sn-PSCs, ensuring consistent film quality over time remains a challenge. Water or oxygen molecule adsorption on tin perovskites is commonly recognized as a key factor contributing to crystal decomposition.^29^ Thus, the incorporation of bulky ammonium cations, such as butylammonium (BA^+^)^30^ and phenylethylammonium (PEA^+^),^31^ has proven effective in reducing moisture ingress at grain boundaries of Sn-based perovskite films, analogous to their function in lead perovskites.^32^ These bulky cations facilitate the transformation of tin-based perovskites from the three-dimensional (3D) architecture into numerous two-dimensional (2D) fragments with hydrophobic characteristics, significantly enhancing the stability of Sn-PSCs.^33^ However, slicing 3D perovskites into partially 2D pieces relies only on weak monodentate chelation, making it challenging to maintain high crystallinity and thereby limiting device performance. Hence, identifying new additives that interact more strongly with Sn-perovskites could further enhance the crystallinity of 2D/3D perovskites, along with simultaneous gains in both PCE and stability.

Here, π-conjugated terpyridine-zinc(II) (ZnTPY) coordination nanosheets (CONASHs), synthesized via a liquid–liquid interfacial method,^34,35^ were used as multidentate chelators to facilitate heterogeneous nucleation of perovskites, thereby strengthening interactions between additives and PEA^+^-containing Sn-perovskite crystals. It has been observed that the incorporation of ZnTPY CONASH supports the formation of (101̅)′ planes in perovskites while concurrently diminishing the prevalence of (202̅)′ facets. This implies that the strong chelation between ZnTPY and the perovskites can effectively restrict the formation of 2D perovskites, even in the presence of bulky PEA^+^ ions. Therefore, the crystallization and orientation of Sn-perovskites are improved, yielding stronger antioxidant passivation and a prolonged photoluminescence (PL) lifetime. Resultantly, the ZnTPY-based Sn-PSC exhibited high operational stability, with a meaningful boost in PCE from 9.14 to 11.59%. This work thus presents a novel additive engineering that enables Sn-perovskites to achieve hydrophobicity while preserving high crystallinity, ultimately leading to high-performance PSCs.

## Results and Discussion

### Synthesis and Fragmentation of ZnTPY CONASHs

**Figure 1:**
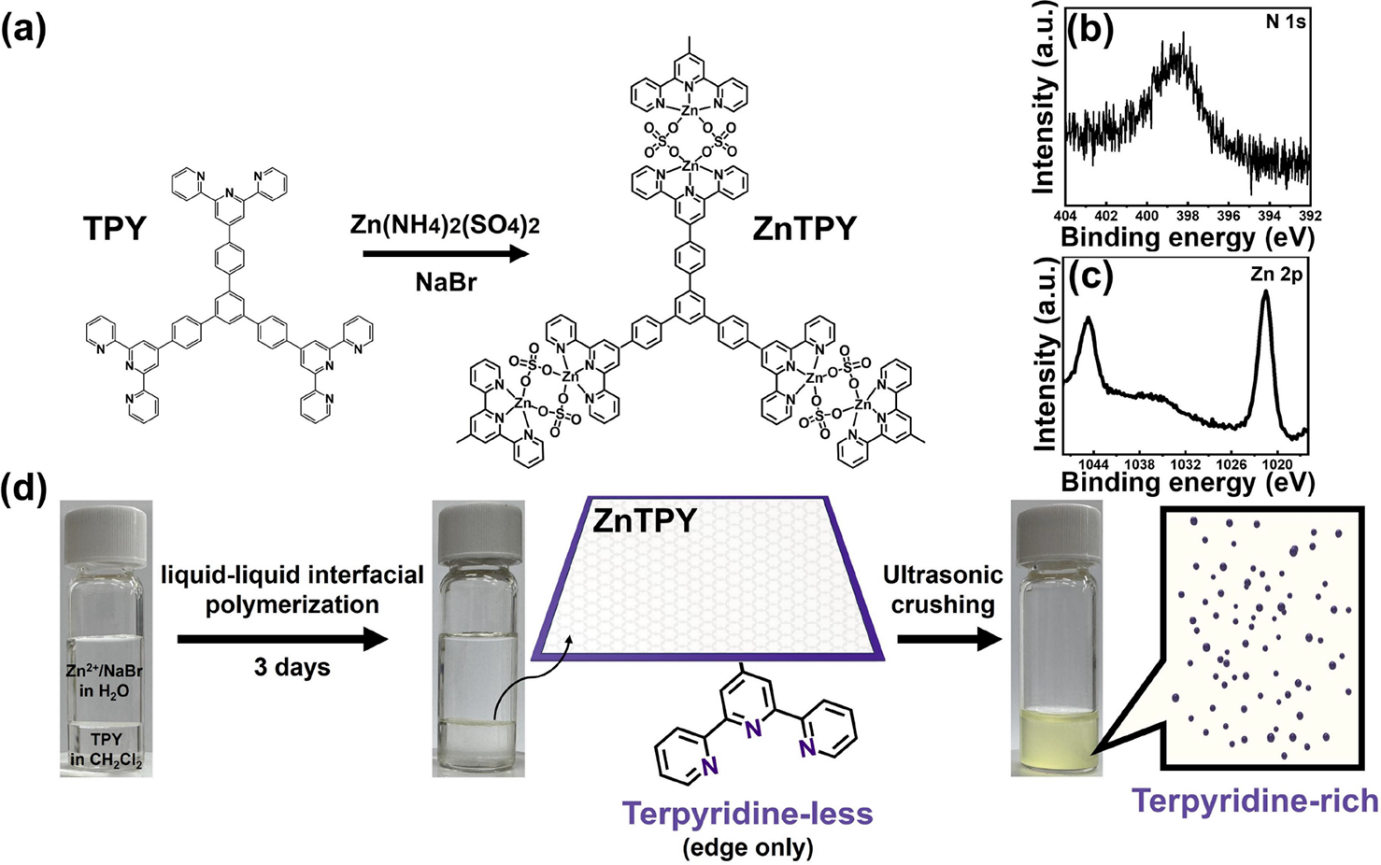
(a) Synthesis pathway of the ZnTPY CONASHs. Figures (b) and (c) present the high-resolution XPS spectra of the N 1s and Zn 2p core levels for the as-synthesized ZnTPY CONASHs. Figure (d) illustrates the liquid/liquid interfacial synthesis process, showing the layered liquid/liquid phases, the reaction after 3 days, and the CONASHs/DMF suspension following ultrasonic fragmentation.

The ZnTPY complex is synthesized via a one-step, bottom-up spontaneous coordination reaction between organic ligand molecules and zinc ions at room temperature, as detailed in our previous report,^34^ resulting in a 2D motif that exhibits the characteristics of metal complexes. The synthesis of CONASHs involves the use of a three-way symmetric 1,3,5-tris[4-(4′-2,2′:6′,2″-terpyridyl)phenyl]-benzene (TPY) as the organic ligand, as illustrated in Figure 1a. Additionally, the Zn(NH_4_)_2_(SO_4_)_2_ salt dissociates in water to yield the inorganic zinc source. Following the reaction, ZnTPY features a structure consisting of repeating units arranged as TPY-Zn(II)-(SO_4_)_2_-Zn(II)-TPY. Figure 1b shows an X-ray photoelectron spectroscopy (XPS) image of a ZnTPY nanosheet, which shows a nitrogen signal in the high-resolution N 1s spectrum at 398.6 eV, indicating that the ZnTPY film is enriched with TPY groups. Continuing to explore higher binding energy positions in the XPS spectrum, satellite doublet peaks in the Zn 2p spectrum are observed at 1021.5 and 1044.7 eV, confirming the presence of Zn(II)-N bonds, as demonstrated in Figure 1c. Therefore, these XPS spectra validate the completion of the coordination reaction between zinc ions and the pyridines of TPY ligands.

In the perovskite precursor solution, we mainly utilized ZnTPY The synthesis of CONASHs involves the use of a three-way symmetric 1,3,5-tris[4-(4′-2,2′:6′,2″-terpyridyl)phenyl]-benzene (TPY) as the organic ligand, as illustrated in Figure 1a, as an additive for the Sn-perovskite. However, the excellent film-forming ability of the as-synthesized CONASHs results in a majority of the reactive TPY groups being coordinated with Zn, which may limit the potential of ZnTPY to enhance the performance of the perovskite. Although bare TPY molecules show a strong chelation effect with the perovskite precursor, they tend to form a gel-like state, which can hinder subsequent solution processing.^34^ To optimize reactivity, coordinating partial TPY ligands with Zn(II) can effectively prevent excessive reactivity while simultaneously increasing the contact area between the additive and the perovskite precursor through the formation of a 2D structure. Figure 1d provides a detailed description of the synthesis steps for the ZnTPY CONASH material, which is abundant in TPY functional groups. The left panel of Figure 1d illustrates a liquid/liquid biphasic polymerization conducted using dual-layer solutions with differing polarities, where the upper layer comprises a high-polarity aqueous solution containing Zn(NH_4_)_2_(SO_4_)_2_ salt and NaBr charge compensator and the lower layer contains TPY dispersed in the low-polarity solvent dichloromethane (DCM). Upon leaving these two phases undisturbed at room temperature for 3 days, the initially transparent interface in the reaction vial gradually turned pale yellow (center of Figure 1d). Figure 1b,c presents XPS spectra that verify the successful formation of ZnTPY CONASHs. After replacing the solvent with dimethylformamide (DMF), we applied ultrasonic agitation to fragment ZnTPY, yielding CONASH particles that are rich in uncoordinated TPY edges (as shown in the right side of Figure 1d, the transmission electron microscopy image, and the corresponding particle size distribution analysis in Figure S1). Next, these tailored ZnTPY CONASHs were added to the perovskite precursor solution to serve as agents for enhancing performance.

### Verification of ZnTPY:SnI_2_ Heterogeneous Nuclei

The PEA_0.15_FA_0.8_MA_0.05_SnI_3_ perovskite photosensitizer used in this study is formed through the reaction of two precursors: organic ammonium iodide and inorganic SnI_2_. Initially, the dissociated I^–^ ions from organic ammonium salts react with SnI_2_ to form octahedral [SnI_6_]^4–^ homogeneous nuclei. These octahedral frameworks subsequently grow into a 3D structure, with organic ammonium ions spontaneously occupying the interstitial spaces between octahedra to form a photoactive perovskite crystal. During this process, the formation of [SnI_6_]^4–^ unit faces a significant energy barrier.^36,37^ When CONASHs are added into the perovskite precursor solution, the uncoordinated TPY sites of incomplete ZnTPY can effectively perform multidentate chelation with SnI_2_ to form [(SnI_6–*x*_)^4–^-ZnTPY] (where *x* represents the number of tin ions coordinated by TPY in the inorganic octahedral structural units within the perovskite crystals) heterogeneous nuclei more readily than I^–^ ions, thereby reducing the crystallization energy barrier of the Sn-perovskite.

**Figure 2:**
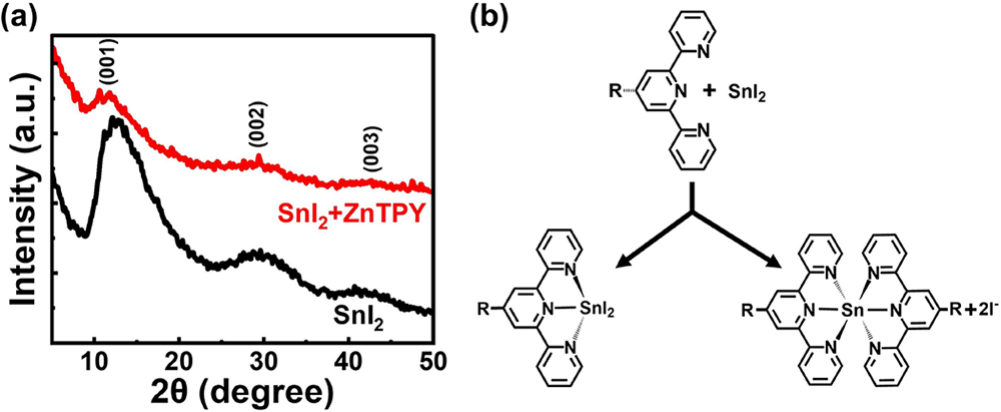
Chelation of SnI_2_ with ZnTPY fragment: (a) XRD signals of ZnTPY:SnI_2_ and bare SnI_2_ films. (b) Two possible mechanisms for the formation of ZnTPY:SnI_2_ complexes.

To verify the formation of heterogeneous nucleation centers, we analyzed the differences in signals from X-ray diffraction (XRD) spectra of SnI_2_ films with and without the addition of ZnTPY. As displayed in Figure 2a, the XRD pattern of the pristine SnI_2_ film exhibits distinct diffraction peaks corresponding to the (001), (002), and (003) crystal planes, confirming the hexagonal phase of SnI_2_.^38^ Compared to the standard sample, the intensity of these diffraction peaks in the ZnTPY CONASH-modified SnI_2_ films was markedly reduced. This change is ascribed to the easy insertion of CONASHs into SnI_2_ aggregates via multidentate chelation, which disrupts the hexagonal lattice structure of SnI_2_.

Based on the findings mentioned above, we can propose two possible coordination pathways. The chemical structures illustrated in Figure 2b suggest that the first pathway involves a single TPY group coordinating with Sn, while the second pathway involves two adjacent TPY units coordinating simultaneously with a single Sn atom. In both scenarios, ZnTPY serves as the multidentate chelator, and this type of chemical interaction is stronger than the reaction between I^–^ and Sn. Therefore, this multidentate coordination complex, which presents a low nucleation energy barrier, will replace the conventional [SnI_6_]^4–^ homogeneous nuclei, resulting in the formation of [(SnI_6–*x*_)^4–^-ZnTPY] heterogeneous nuclei (hereafter referred to as ZnTPY:SnI_2_). Moreover, the chemical composition of ZnTPY:SnI_2_ complexes was analyzed via Fourier transform infrared (FTIR) spectroscopy. As shown in Figure S2, the pristine ZnTPY displayed a transmission band at 1645 cm^–1^, attributed to the C=N stretching mode within its pyridyl framework. This site acts as the primary coordination site for TPY to bind with Sn(II) ions. Following Sn-perovskite crystal incorporation, the characteristic C=N vibrational signal migrated to 1657 cm^–1^. Such a shift implies strengthened electron delocalization due to metal–ligand coordination, consistent with the emergence of ZnTPY:SnI_2_ hybrid complexes.^34^

### Characterization of Sn-Perovskites Modified with ZnTPY CONASHs

**Figure 3:**
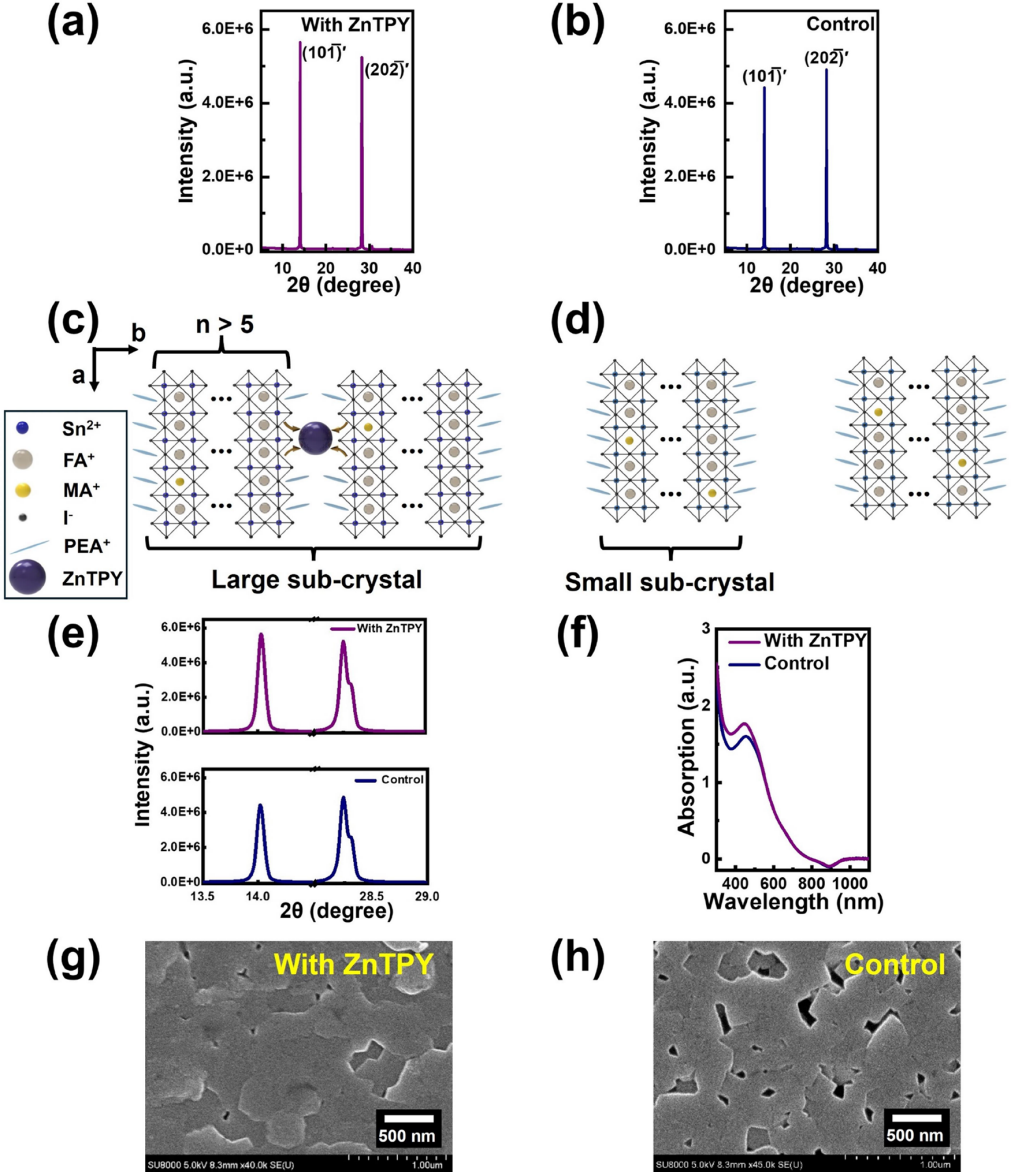
XRD patterns of PEA_0.15_FA_0.8_MA_0.05_SnI_3_ films: (a) with and (b) without ZnTPY CONASHs. (c) Schematic structure of the ZnTPY-based PEA_0.15_FA_0.8_MA_0.05_SnI_3_ crystal and (d) bare PEA_0.15_FA_0.8_MA_0.05_SnI_3_ crystal. (e) Enlarged XRD peaks for the (101̅)′ and (202̅)′ planes. (f) Absorption spectra of Sn-perovskite films with and without ZnTPY. SEM images of Sn-perovskite films: (g) with ZnTPY additives and (h) without ZnTPY.

To investigate the structural changes imparted by the ZnTPY:SnI_2_ heterogeneous nuclei on Sn-perovskite crystals, we conducted a detailed analysis using the X-ray diffraction (XRD) technique. Herein, ZnTPY CONASHs are dissolved in the PEA^+^-containing perovskite precursor solution at a concentration of 0.075 mg/mL. Subsequently, ZnTPY-seeded PEA_0.15_FA_0.8_MA_0.05_SnI_3_ perovskite films are fabricated using an antisolvent spin-coating method (for details, refer to the Experimental Section). Generally, the introduction of bulk PEA^+^ ions spontaneously cleaves the 3D Sn-perovskite crystals into 2D few-layered components (*n* < 5). If the stoichiometric ratio of PEA^+^ is insufficient, the Sn-perovskite structure develops to its 3D analog, specifically a multilayered 2D (*n* > 5)/3D hybrid structure. In this scenario, the structure of the 2D/3D perovskite increasingly approximates a 3D configuration. This indicates that the addition of a small amount of PEA^+^ to the Sn-perovskite results in the XRD signal manifesting at an angle comparable to that observed in the 3D structure.^33^ From our study, the (101̅)′ and (202̅)′ facets observed in the quasi-cubic 2D/3D PEA_0.15_FA_0.8_MA_0.05_SnI_3_ perovskite exhibit similar angles, with the two peaks differing by approximately 14 and 28°, respectively, when compared to the (100) and (200) planes of the cubic 3D Sn-perovskite crystals,^39,40^ as illustrated in Figure 3a. The addition of PEA^+^ caused no significant shifts in the diffraction angles of these two pairs of crystal planes, suggesting that the in-plane lattice parameters along the a and c axes remain undistorted. With the addition of ZnTPY:SnI_2_ heterogeneous nuclei, the quasi-cubic 2D/3D Sn-perovskite also exhibits the same crystal planes (Figure 3b). Interestingly, the addition of ZnTPY results in a stronger peak intensity for the (101̅)′ plane compared to the (202̅)′ facet, indicating a reduction in the periodicity of the PEA_0.15_FA_0.8_MA_0.05_SnI_3_ perovskite crystal along the *b*-axis, as shown in Figure 3c. This directly confirms that the introduction of ZnTPY:SnI_2_ adducts leads to the formation of strong chelation between ZnTPY and Sn-perovskites, effectively preventing PEA^+^ from rapidly cleaving crystals. In contrast, Figure 3d illustrates how the incorporation of the bulky PEA^+^ molecule expands the 3D structure of the PEA_0.15_FA_0.8_MA_0.05_SnI_3_ crystal, disrupting continuity along the *b*-axis. Figure 3e demonstrates that the Sn-perovskite containing ZnTPY:SnI_2_ displays improved crystallinity, supporting the claim that the PEA^+^-based perovskite has been effectively reconstructed. Additionally, XPS spectra were employed to investigate the effect of crystallinity optimization in Sn-perovskite crystals on changes in chemical composition (Figure S3). To estimate the oxidation state of Sn, we analyzed the characteristic peaks of Sn^2+^ and Sn^4+^, which were split into two satellite signals, with binding energies of approximately 485.5 and 493.9 eV corresponding to the 3d_5/2_ and 3d_3/2_ states of Sn^2+^, respectively, while 486.3 and 494.7 eV correspond to the 3d_5/2_ and 3d_3/2_ states of Sn^4+^.^5^ It was observed that the proportion of Sn^4+^ in the Sn-perovskite with the ZnTPY additive decreased from 14.38 to 9.57%. After peak integration, the proportion of Sn^4+^ in the ZnTPY-modified Sn-perovskite decreased from 14.38 to 9.57%. As a multidentate ligand, ZnTPY can chelate with Sn^2+^ to form a coordination complex, thereby inhibiting the oxidation of the Sn-perovskite film. This interaction improves the defect chemistry of the Sn-perovskite, leading to enhanced film quality. In sum, ZnTPY CONASHs can effectively retain the hydrophobic macroion PEA^+^ within the crystal and maintain the high-quality quasi-cubic 2D/3D structure of tin perovskite through strong interactions with SnI_2_.

The alteration in the crystal structure upon the inclusion of ZnTPY:SnI_2_ heterogeneous nuclei was further validated through analyses using UV–vis absorbance spectroscopy and scanning electron microscopy (SEM). As presented in Figure 3f, the enhanced band-edge absorption of Sn-perovskite crystals within the wavelength range of 310–530 nm signifies improved crystallinity and orientation of Sn-perovskites^41^ that have been treated with ZnTPY:SnI_2_ adducts. Moreover, this observation reflects the suppression of the formation of the 2D less-layered perovskite architecture.^42^ Thus, as demonstrated in the SEM image, the growth of Sn-perovskites with *b*-axis orientation promotes the formation of compact and smooth films (Figure 3g). In contrast, bare PEA_0.15_FA_0.8_MA_0.05_SnI_3_ films exhibit significant roughness, characterized by pinholes and voids resulting from uncontrolled crystal growth (Figure 3h). The enhanced morphology of the Sn-perovskite film, attributed to the presence of ZnTPY:SnI_2_ heterogeneous nuclei, can be further elucidated by the uncoordinated TPY sites of ZnTPY CONASHs in the perovskite precursor solution. These TPY-terminated groups promote chelation with SnI_2_, leading to the formation of [(SnI_6-x_)^4–^-ZnTPY] heterogeneous nuclei with low activation energies, thereby enhancing intuitive morphological optimization.

### Photovoltaic Performance

**Figure 4:**
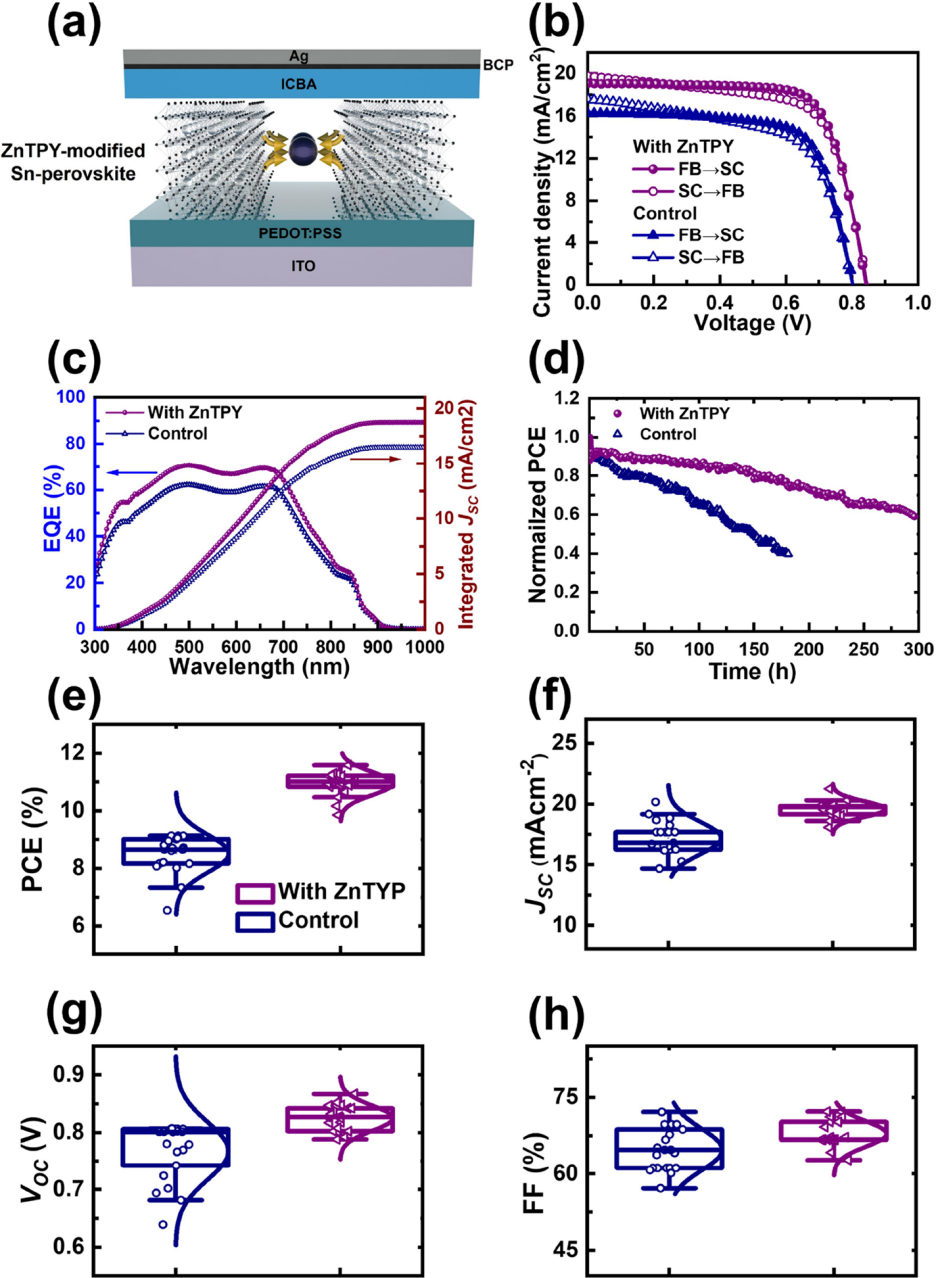
(a) Schematic representation of Sn-PSC device architecture, incorporating ZnTPY CONASHs as heterogeneous nucleation sites. (b) Reverse (FB→SC) and forward (SC→FB) *J–V* characteristics measured under AM 1.5G illumination (100 mW cm^–2^) for Sn-PSCs with and without the inclusion of ZnTPY complex. (c) EQE spectrum and corresponding integrated *J*sc curve of the highest-performing Sn-PSCs with and without insertion of ZnTPY CONASHs. (d) The operational stability of Sn-PSCs with and without ZnTPY integration was evaluated under maximum power point tracking conditions using encapsulated devices in ambient air with a relative humidity of 30–40%. Statistical analysis of (e) PCE, (f) *J*sc, (g) *V*oc, and (h) FF for Sn-PSCs with and without ZnTPY CONASHs.

To evaluate the impact of crystallinity and morphological optimization on photovoltaic performance, we examined ZnTPY-incorporated perovskite sensitizers in Sn-PSCs with an architecture of indium tin oxide (ITO)/poly(3,4-ethylenedioxythiophene):polystyrenesulfonate (PEDOT:PSS)/perovskite/indene-C_60_ bisadduct (ICBA)/bathocuproine (BCP)/Ag, as schematically depicted in Figure 4a. In the device structure, PEDOT:PSS, ICBA, and BCP serve as the hole transporter, electron transporter, and hole-blocking layer, respectively. These thin films play a crucial role in the dissociation of excitons within the Sn-perovskite and enhance the transport of the resulting free charge carriers, ultimately mitigating electron–hole recombination in PSCs. Figure 4b presents the current density–voltage (*J–V*) characteristics of Sn-PSCs both without and with ZnTPY CONASHs, measured under simulated AM 1.5G illumination at the intensity of 100 mW/cm^2^. The comprehensive *J–V* parameters are outlined in Table 1. The incorporation of ZnTPY leads to the formation of highly crystalline and smooth films, resulting in the highest PCE observed. The best PCE achieved with the incorporation of ZnTPY as additives in the perovskite layer is 11.59%, accompanied by a short-circuit current density (*J*sc) of 19.06 mA/cm^2^, an open-circuit voltage (*V*oc) of 0.842 V, and a fill factor (FF) of 72.19%. These values surpass those of the control PSC device, which exhibited a PCE of 9.14%, a *J*sc of 16.25 mA/cm^2^, a *V*oc of 0.807 V, and an FF of 69.70%, implying that the ZnTPY CONASHs is an ideal additive for Sn-PSCs. The external quantum efficiency (EQE) spectrum allows for an analysis of the details behind the enhancement of *J*sc, as shown in Figure 4c. The findings indicate that incorporating ZnTPY enhances photocurrent across the 300–850 nm range, effectively covering the entire visible spectrum. This advancement is primarily attributed to high crystallinity, compact morphology, and defect suppression. The reduction of defects (vacancies) helps mitigate hysteresis resulting from the polarization of the perovskite layers caused by the migration of iodide ions.^43^ Hence, in Sn-PSCs constructed with ZnTPY CONASHs, a reduced *J–V* hysteresis is observed across various scanning directions (Figure 4b). Additionally, the ZnTPY-containing tin perovskite, which already incorporates hydrophobic PEA^+^ macromolecular, exhibits more complete crystallization compared to the pristine PEA_0.15_FA_0.8_MA_0.05_SnI_3_ perovskite. This modification is anticipated to enhance its resistance to the adhesion of water and oxygen, thereby increasing stability. We thus assessed the long-term operational stability of Sn-PSCs with device encapsulation by comparing those with and without ZnTPY CONASHs through maximum power point tracking, as shown in Figure 4d. After 180 h, the PCE of the reference cell decreased to below 40% of its initial value, whereas the ZnTPY-based Sn-PSC retained approximately 60% of its original PCE after ∼300 h. These results confirm that ZnTPY CONASHs contribute to enhancing the stability of Sn-PSCs.

**Table 1:** Summarized Parameters of Sn-PSCs with and without ZnTPY CONASHs Were Measured under Simulated AM 1.5G Illumination at 100 mW cm^–2^^a^

devices	*J*_SC_ (mA cm^–2^)	*V*_OC_ (V)	FF (%)	PCE (%)
with ZnTPY (FB→SC)	19.06	0.842	72.19	11.59 (10.95 ± 0.40)
with ZnTPY (SC→FB)	19.83	0.848	66.70	11.21
control (FB→SC)	16.25	0.807	69.70	9.14 (8.51 ± 0.65)
control (SC→FB)	17.68	0.801	61.12	8.66

a The average values, along with the standard deviations (shown in brackets), were statistically calculated from data collected from 21 devices across 4 distinct batches.

Moreover, we noted that the ZnTPY-planted Sn-PSCs displayed a statistically higher average PCE of 10.95 ± 0.40% with a narrower distribution compared to the control device, indicating reproducible superior performance, as illustrated in Figure 4e–h (detailed parameters) and summarized in Table 1 (PCE values).

### Photodynamic Analysis

**Figure 5:**
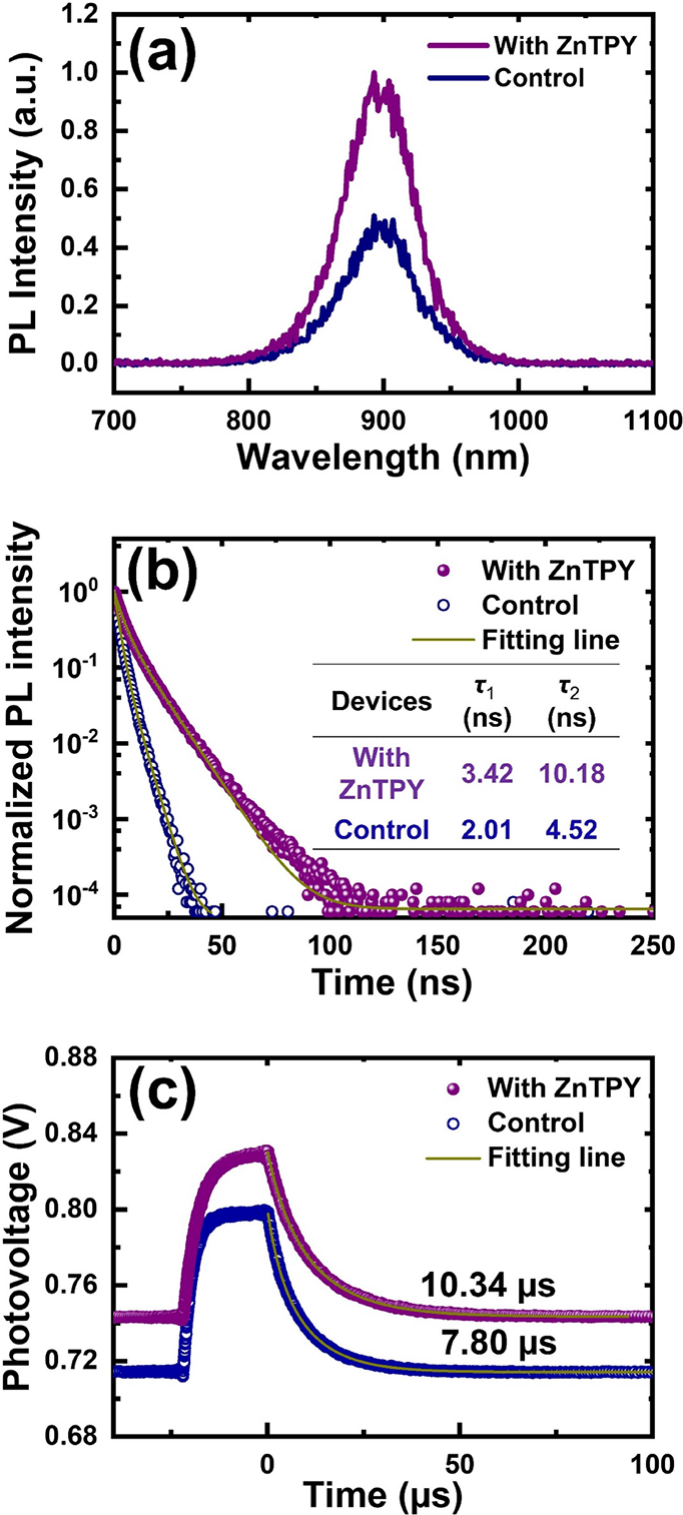
(a) Steady-state PL, (b) TRPL, and (c) TPV decay curves of Sn-PSCs with and without ZnTPY additive.

Next, we investigated the correlation between enhanced photovoltaic performance and improved crystallinity of Sn-perovskite by analyzing photodynamic behaviors. Steady-state photoluminescence (PL) measurements revealed that perovskite films without any transport layers do not exhibit quenching, instead, only charge-carrier recombination is observed. Thus, the intensity of recombination is directly related to the quantity of photogenerated carriers generated inside the perovskite photoabsorber.^44^ As illustrated in Figure 5a, the PL intensity of the ZnTPY-doped perovskite film is significantly higher than that of the bare Sn-perovskite, confirming that the introduction of ZnTPY CONASHs into the PEA_0.15_FA_0.8_MA_0.05_SnI_3_ perovskite enhances photocurrent generation. Typically, perovskites with better crystallinity and fewer defects exhibit an increase in photocurrent.^45^ Therefore, the PL results demonstrate a trend that aligns with the results presented in Figure 3, indicating that ZnTPY CONASHs can enhance the generation of photocarriers by improving the crystallinity of the Sn-perovskite, ultimately leading to enhanced solar cell performance. For a more comprehensive analysis, the dynamics of photogenerated carriers within the tin perovskite can be assessed using time-resolved PL (TRPL) decay curves, as seen in Figure 5b. The TRPL analysis revealed a biexponential decay in the recombination dynamics, with each curve being deconvoluted into two stages characterized by distinct lifetimes. The inset table of Figure 5b lists the TRPL lifetimes, with τ_1_ and τ_2_ representing the fast and slow decay lifetimes of photocarriers following prolonged excitation, where τ_1_ is associated with surface recombination and τ_2_ corresponds to recombination within the interior of the pristine perovskite.^46^ Clearly, the introduction of CONASHs into Sn-perovskite films yielded longer τ_1_ (3.42 ns) and τ_2_ (10.18 ns) values compared to bare films (τ_1_ = 2.01 ns and τ_2_ = 4.52 ns), indicating that charge-carrier recombination is taking place not only at the crystal surface but also within its interior. This implies that ZnTPY enhances the morphology of Sn-perovskite from the interior outward, thereby thoroughly improving the generation of photocurrent. In addition to investigating the photodynamics of the perovskite film, we further examined the impact of ZnTPY CONASHs on enhancing Sn-PSC device performance. We applied laser pulses to modulate the *V*oc and further measured the decay of transient photovoltage (TPV) signals to conduct device-level photodynamic analysis. Figure 5c illustrates that the charge-recombination lifetime decreased from 10.34 μs in the ZnTPY CONASH-treated PSC to 7.80 μs in the reference cell. The longer carrier recombination lifetime observed in the PSC containing CONASHs suggests that the chelation between the terminal TPY groups of fragmented ZnTPY and defective perovskite crystals mitigates surface defects, thereby decreasing trap-assisted recombination.^47,48^

## Conclusions

In summary, this study demonstrates that ZnTPY CONASHs are a promising additive for the formation of ZnTPY:SnI_2_ heterogeneous nuclei, effectively repairing quasi-cubic 2D/3D Sn-perovskite crystals affected by the insertion of bulky PEA^+^ molecules. The experimental results indicate a significant reduction in the 2D phase, resulting in an overall enhancement of crystallinity. The incorporation of ZnTPY-modified tin perovskite as the photoactive layer in PSC devices leads to simultaneous improvements in both PCE and operational stability. Thus, π-conjugated CONASHs offer an innovative crystallization strategy for Sn-perovskites and demonstrate broad applicability for enhancing the performance of other environmentally friendly PSCs.

## Experimental Section

### Synthesis of the ZnTPY CONASH

The ZnTPY CONASH was synthesized at a liquid–liquid interface within a perfluoroalkoxy alkane (PFA) container, following a detailed procedure outlined in the main article. Initially, a TPY solution (5.5 mg of TPY dissolved in 11 mL of degassed dichloromethane) was added to the PFA container, and then 11 mL of deionized water was gently layered on top. Next, a zinc(II) solution [comprising 34.28 mg of Zn(NH_4_)_2_(SO_4_)_2_·*x*H_2_O (Thermo Fisher Scientific) and 1.1 mg of NaBr (Sigma-Aldrich, ≥99.0%) dissolved in 11 mL of degassed deionized water] was carefully introduced to the upper layer. After 3 days, the solution was entirely removed, leaving the ZnTPY CONASH. This product was thoroughly washed with ethanol to eliminate residual impurities. Finally, 1 mL of DMF was added to the ZnTPY CONASH, followed by ultrasonic treatment for 1 h to yield ZnTPY fragments.

### Materials Used in Tin Perovskite Solar Cells

All chemicals were obtained from commercial suppliers as specified and, unless stated otherwise, were used without further purification. The materials included formamidinium iodide (FAI, TCI), methylammonium iodide (MAI, ≥ 99%, Sigma-Aldrich), SnI_2_ (99.999%, Sigma-Aldrich), SnF_2_ (99%, Sigma-Aldrich), and tin nanopowder (<150 nm particle size, ≥99%, Sigma-Aldrich). Additional chemicals included PEDOT:PSS (Clevious, A14083), ICBA (99% purity, TCI), and BCP (99% purity, Sigma-Aldrich), all used as received.

### Device Fabrication

The precleaned ITO glasses were first treated with UV-ozone for 15 min. Subsequently, a PEDOT:PSS hole transport layer, diluted to 50% with methanol, was spin-coated onto the ITO substrate at 4000 rpm for 30 s and annealed at 150 °C for 20 min in ambient conditions. After this step, the ITO/PEDOT:PSS was transferred into a nitrogen-filled glovebox. The Sn-based perovskite precursor solution (0.85 M) was prepared by dissolving FAI, MAI, PEAI, SnI_2,_ and SnF_2_ in dimethyl sulfoxide in a 0.8:0.05:0.15:1:0.1 molar ratio, along with 5 mg of Sn powder. For the ZnTPY additive, a ZnTPY/DMF solution was added to the precursor solution at a volume ratio of 0.5%. The perovskite precursor solution containing ZnTPY CONAHSs was spin-coated with a 2-s ramp-up to 6000 rpm, followed by 90 s at this constant speed. At the 60-s mark during the spin-coating, 0.15 mL of chlorobenzene (CB) was added as an antisolvent. The films were then annealed at 90 °C for 15 min to induce crystallization. For the electron transport layer, ICBA (18 mg/mL in CB) was spin-coated in a two-step process: 1000 rpm for 30 s and then 5000 rpm for 5 s, followed by annealing at 75 °C for 5 min. Next, a BCP (1 mg/mL in isopropanol) hole-blocking layer was deposited by spin-coating at 5000 rpm for 20 s with a 2-s ramp-up, and subsequently annealed at 70 °C for 5 min. Finally, a 150 nm layer of Ag was thermally deposited as the metal electrode. The fabricated devices, with an active area of approximately 0.26 cm^2^, were sealed with UV-curable resin prior to measurement in ambient conditions.

### Device Characterizations

For growth characterization, in NIMS Battery Research Platform facilities, XRD patterns of fabricated Sn-HaP films were collected using an advanced X-ray diffractometer (Rigaku SmartLab, CuK_α_ radiation, λ = 1.54050 A). XPS spectra were obtained using a Versa Probe II (ULVAC-PHI, Japan). Perovskite film samples for XPS measurements were prepared in an N_2_-filled glovebox and transferred to the XPS chamber through an N_2_-filled transfer vessel to avoid oxygen contamination. XPS with a nonmonochromatic source was carried out (Al Kα; 1486.6 eV, spot size 10–300 μm) at a pass energy of 187.85 eV (1.5 eV step size) for the survey scan and pass energy 46.95 eV (0.1 eV step size) for the fine scan with spot size 100 μm. The XPS spectra were calibrated with a binding energy of 284.8 eV for C 1s.

In NIMS Namiki foundry research facilities, the morphology of films and cross-sectional images were taken by a high-resolution SEM at 5 kV accelerating voltage (Hitachi, S-4800). The PL spectra were collected using a micro-PL spectrometer [HORIBA, LabRamHR-PL NF(UV-NIR)] ∼532 nm laser diode (10 mW cm^–2^) as an excitation source. The carrier lifetimes were measured with a fluorescence lifetime spectrometer (Quantaurus-τ from Hamamatsu-Photonics K.K., C11367) equipped with a ∼405 nm laser diode (typical peak power of 400 mW) at a 200 kHz repetition rate. The absorption spectra films were measured using a UV–vis-NIR spectrometer (UV-2600i, Shimadzu). The absorption spectra and PL spectra of various films were measured using a UV–vis-NIR spectrometer (UV-2600i, Shimadzu). The *J–V* curves were measured at a scan rate of 0.05 V/s under 1 sun with an AM1.5G spectral filter (100 mW/cm^2^) coupled with an MPPT system (Systemhouse Sunrise Corp.). The light intensity was calibrated by a silicon (Si) diode (BS-520BK). For the stability test, the devices were measured at MPPT conditions and at ambient conditions. The *J–V* curves were measured with a scan rate of 0.05 V/s under 1 sun with an AM 1.5G spectral filter (100 mW cm^–2^) coupled with an MPPT system (Bunkoukeiki Corp. The EQE spectra were obtained using a spectrometer (SM-250IQE, Bunkokeiki, Japan). The transient photovoltage data were measured using a commercial PAIOS system (PAIOS V.4.3). A pulse intensity was used to induce a spike in photovoltage.

## Supporting Information Available

TEM image, FTIR spectra, and XPS spectra (PDF)
