# Supplementary material for: Coordination Nanosheets Stabilizing Efficient Tin-Based Perovskite Solar Cells
Source: ACS Appl Mater Interfaces. 2025 Apr 24;17(18):26813–22. doi: 10.1021/acsami.5c05011 (PMC12067372; doi:10.1021/acsami.5c05011)
Supplement: Supplementary file 1 — am5c05011_si_001.pdf [file am5c05011_si_001.pdf]

Supporting Information

Coordination nanosheets stabilizing efficient tin-based perovskite solar cells

Dhruba B. Khadka[1,*], Yan-Chen Kuo[2], Yi Zhen Li[2], Muhammad Waqas[2], You-Jia Xu[2], Masatoshi Yanagida[1], Hiroshi Nishihara[3], Kazuhito Tsukagoshi[4], Mitch M. C. Chou[5], Yasuhiro Shirai[1] and Ying-Chiao Wang[2,*]

[1]Photovoltaic Materials Group, Center for GREEN Research on Energy and Environmental Materials, National Institute for Materials Science (NIMS), 1-1 Namiki, Tsukuba, Ibaraki 305-0044, Japan

[2]Department of Materials and Optoelectronic Science, National Sun Yat-sen University, Kaohsiung, 804, Taiwan, R.O.C.

[3]Research Institute for Science and Technology, Tokyo University of Science, 2641 Yamazaki, Noda, Chiba 278-8510, Japan

[4]WPI International Center for Materials Nanoarchitectonics (WPI-MANA), National Institute for Materials Science (NIMS), 1-1 Namiki, Tsukuba, Ibaraki 305-0044, Japan

[5]Academy of Innovative Semiconductor and Sustainable Manufacturing, National Cheng Kung University, Tainan, 70101, Taiwan, R.O.C.

*Correspondence and requests for materials should be addressed to D. B K. (email: KHADKA.B.Dhruba@nims.go.jp) and Y.-C. Wang (email: ycwang@mail.nsysu.edu.tw)

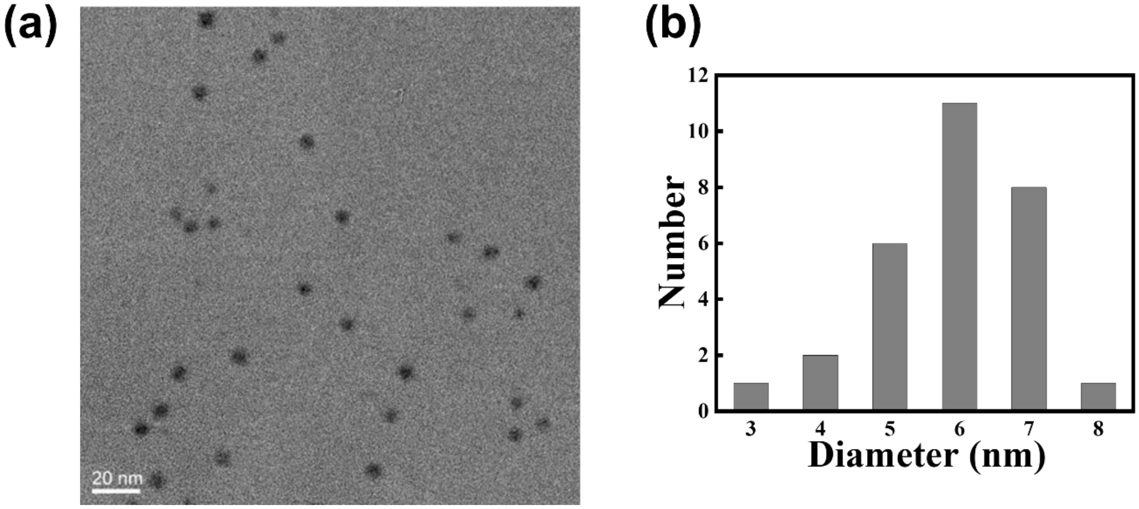

Figure S1. (a) TEM image and (b) corresponding particle size distribution analysis of ZnTPY CONASHs after fragmentation.

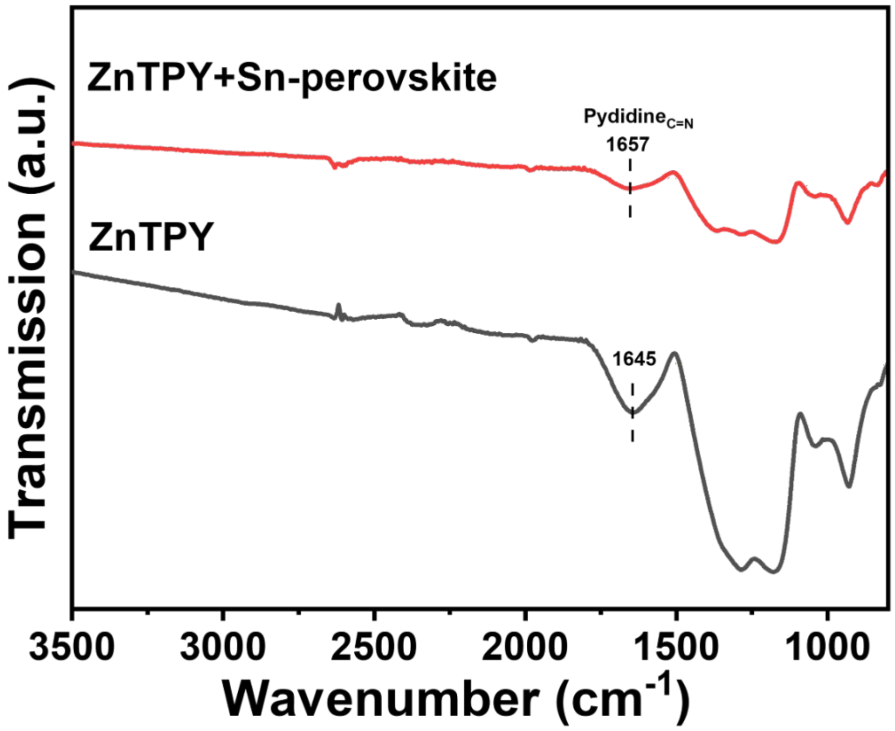

Figure S2. FTIR spectra of ZnTPY and the ZnTPY-Sn perovskite hybrid.

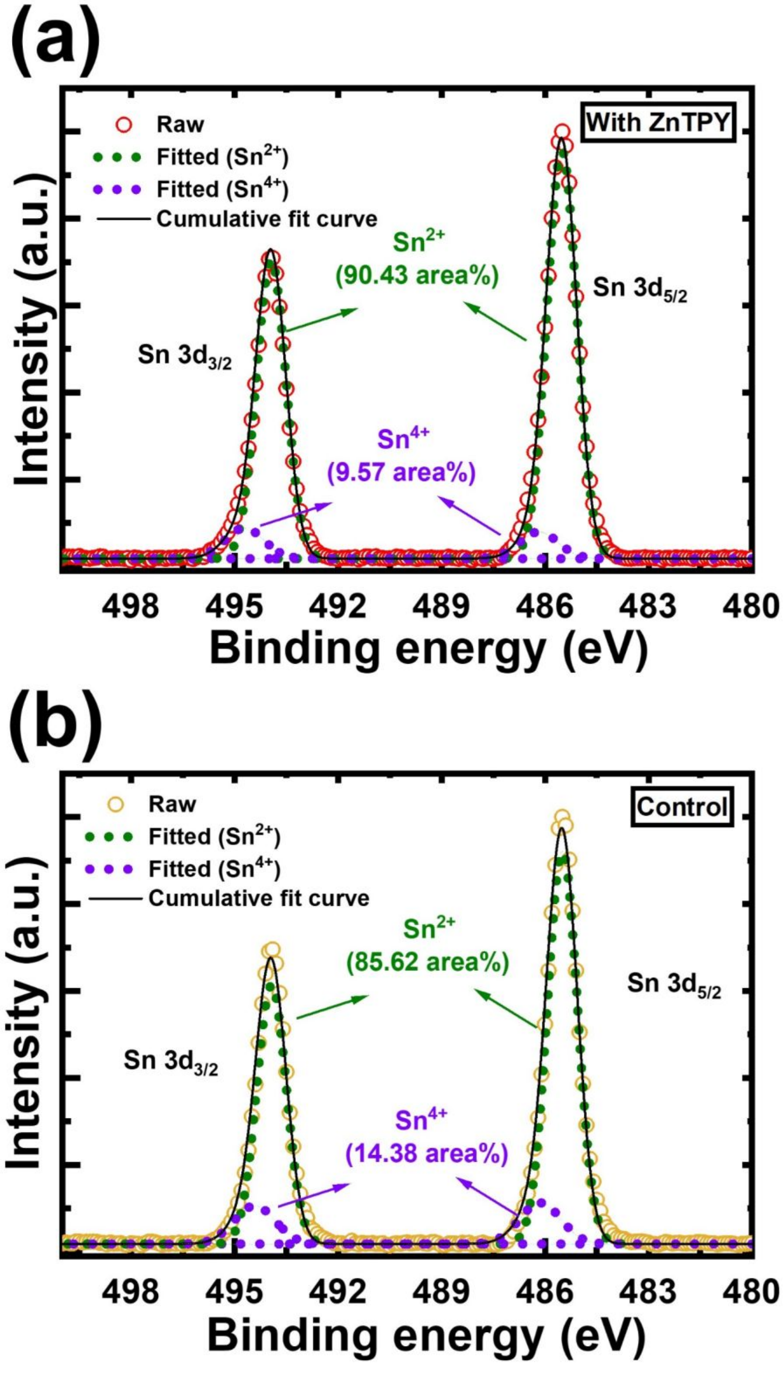

Figure S3. High-resolution XPS spectra of the Sn 3d core levels in Sn-perovskites (a) with and (b) without ZnTPY CONASHs.
